# Ether

**Published:** 1881-05

**Authors:** Laurence Turnbull

**Affiliations:** Aural Surgeon to Jefferson Medical College Hospital, Philadelphia


### Independent Practitioner.

VOL. II.——*May, 1881.*——No. 5.

ARTICLE I.

(Continued from page 170.)

### ETHER.

BY LAURENCE TURNBULL, M. D.,
Aural Surgeon to Jefferson Medical College Hospital, Philadelphia.

Ether we consider par excellence the most safe anæsthetic agent for long operations yet discovered ; and yet, as we stated in our introductory remarks, there has not yet been discovered an absolutely safe anæsthetic, and we doubt if such an agent ever will be discovered. Ether should not be employed without a selection of the cases to which it is truly applicable.

There are a few exceptional cases in which we have noticed that there occurs under the very free use of even a pure ether a partial paresis of the respiratory muscles, with an excessive secretion of mucus, which has a tendency to cause laryngeal spasm if the ether is not mixed with the vapor of warm water. If ice is freely used in the mouth some time prior to the use of the anæsthetic, or a solution of bromide of potassium applied by the spray apparatus, they will tend to prevent this spasm. In all our operations recently we have employed the hot water on the sponge so as to mix the two vapors before being received into the lungs. Again, since the almost universal use of Squibbs ether, which contains much less water than the ordinary ethers, these peculiar symptoms of obstructed respiration have been noticed, and a few instances of sudden death have occurred ; one in the hands of Dr. Levis. It may be, as was first suggested by Dr. Dawson of England, that the cold caused spastic contraction of the capillaries.

If so, the warm water vapor will relieve this spasm. Our opinion has always been that we use too much ether and fill the lungs so full that not enough air containing oxygen is allowed to enter, and no exchange of the carbonic acid in the lungs with the oxygen of the atmosphere takes place. This is followed by asphyxia, and death may result from this cause. We must also bear in mind that ether will produce, if carried to its extreme poisonous anæsthetic effects, a true toxicological result: a paralysis of conscious sensation; paralysis of the movement of voluntary muscles; paralysis of respiration, circulation, and, finally, paralysis of the heart and the vasomotor nerves occur. Respiratory paralysis is produced by ether when the circulation and blood pressure remain within the limits compatible with life. Sometimes the vascular pressure increases, sometimes it decreases, but it is always sufficiently high to allow the exchange of the carbonic acid gas with the oxygen of the atmosphere.

Vascular succeeds respiratory paralysis when ether is administered. The reverse takes place with chloroform. Frequently an amount of this latter anæsthetic agent which would not be sufficient to produce respiration, may suffice to bring on vascular paralysis. Under these conditions should vascular paralysis last over thirty seconds, artificial respiration is useless.

Dr. Kappeler states, on page 167: " My observations on the pulse during ether-narcosis, with the aid of the sphygmograph, 10 in all, of which 7 show that in this small number appear three in which the tracing shows either no change at all, or one scarcely appreciable, although the patient was in each case etherized to complete anæsthesia, the extinction of reflex excitability of the cornea and to contraction of the pupil; a condition that, found in none of the 25 cases of chloroform-narcosis of which sphygmographic tracings had been taken. Seven times in all, and by the tracings here reproduced, 4 times does the tracing of profound ether-narcosis differ in no manner from that of profound chloroform-narcosis. Here, as there, a club-like blunting of the apex of the tracing, flattening of the great incisure, and several times distinct anacrotism. On the whole, the elevation of the receding beat is more distinctly marked in the ether tracings than in the chloroform tracings, and repeatedly we find a tendency to discrotism in the ether tracings.

Deaths, it is stated, have occured from ether in considerable number in England, almost every one of which has been owing to the use of impure ether. We have given the whole number of deaths from ether, 18, on the authority of an English writer, Dr. Dawson, and they are very few; and if the gentleman had had much opportunity of experimenting on animals, he would have soon found out how dangerous chloroform was to animal life, and how freely he could use ether. This was also proven to us by Schiff, and many other distinguished physiologists whose laboratories we visited in the year 1879; this is also the opinion of our friend the late Professor James Aitken Meigs and Professor Dalton of New York. There are a few peculiarities to be noted with regard to ether on ship board, or in very hot climates where it cannot be used on account of the high temperature; and in some very rare cases, when it is absolutely necessary to use an anæsthetic and ether will not produce its effects, we must use the material with all the precautions that are necessary, looking for death at any moment.

The cause of secondary death from ether is almost invariably reduction of temperature of the skin after its use, with effusion into the lungs. We have before stated that if the patient be carefully guarded from becoming chilled, and no whisky, brandy, or alcohol administered, which will cause filling up of the lungs with mucus, the number of deaths are so small with ether that it ranks in safety next to nitrous oxide. Dr. Kappeler only gives 13 deaths. We repeat the statement that ether, when pure and not mixed, always gives warning as in cases Nos. 6 and 8. On the list of deaths, eighteen to twenty all told, from ether (see p. 40), as stated by our reviewer, some of these seem to have been as sudden as any under chloroform, as far as can be judged from the brief reports. If the doctor had but turned to page 39 he would have found the cause. It was "Robbin's ether" that was employed, and Dr. Richardson says it was not a pure ether, but a mixture of amyl hydride and anhydrous ether, being of a low boiling point and specific gravity, and it is directly dangerous when inhaled. As to case No. 8, a patient at the Homœopathic College, New York, was etherized for an operation upon a necrosed jaw. The sudden blueness and the ceasing of respiration was owing to the upright position of the patient and blood getting into the trachea and causing suffocation. This the post mortem subsequently revealed.

### Deaths from the Inhalation of Ether.

The Canada *Lancet* (December, 1879) reports a case of sudden death from the inhalation of ether, which occurred at an asylum but a few weeks previously. Scarcely an ounce of ether was administered for the purpose of extracting a tooth. The coroner's jury found that the death was due to paralysis of the heart, caused by the inhalation of ether. There must have been chloroform with it, or an English mixed ether. A case is reported at Providence (*Boston Med. and Surg. Journal*, Jan. 15, 1880) in which the ether was given to facilitate the diagnosis of an injury to the hip. After the inhalation, for about fifteen minutes, the man suddenly ceased to breathe. The autopsy showed effusion of serum beneath the arachnoid, valvular disease of the heart, and cystic degeneration of the kidneys (the latter was evidently the chief cause of death).

Death from the secondary effects of ether,* occurring at the Cincinnati Hospital, (Ohio) fully three hours and a half after the onset of the first bad symptoms, and the same length of time after the last inhalation of ether. The individual, a Mrs. Henderson, age 43, entered the hospital September 26, 1880; she had been suffering with this abscess, and frequent symptoms of septicaemic poisoning, from June 14th until her admission to the hospital. During that time she had been under the care of Dr. King, who attended her in confinement on Aug. 13th. A fortnight later the doctor opened an abscess at the outer side of the knee, which was followed by septic poisoning; shortly after this, she was sent to the hospital and came under the care of Dr. D. She was evidently depressed at the thought of being in a hospital. The matter discharged from the abscess was of a dark mucus and unhealthy character. Tonics, food and stimulants were freely given, and the day after admission she was brought into the amphitheatre, and, under the influence of ether, Dr. D. enlarged the opening and found no denuded bone; washed out the abscess with a strong solution of chloride of zinc to purify the cavity; antiseptic dressings with a drainage tube were then applied. The day following this operation the amount of discharge was *great*, mixed with a good deal of *blood*. The quantity of the discharge diminished somewhat after first few days, and

*A clinical lecture delivered at the Cincinnati Hospital by N. P. Dandridge, M. D., the *Cincinnati Lancet and Clinic*, Oct. 30th, 1880.

its character decidedly improved, but it continued considerable, so that he resolved to again explore the abscess; the operation being again done under ether. During the administration no bad symptoms were noticed. He passed a probe through the existing opening to find the direction of the cavity and determine the point of a counter incision. He then came in contact with a considerable portion of the femur which was laid bare and necrosed and almost completely encircled by the abscess. Dr. D. determined to make no further incision until he had obtained the consent of the patient and consultation of the staff. The antiseptic dressing was again applied. The examination had been made under the antiseptic spray.

The consent was obtained and the staff was notified to examine the patient while on the operating table, so if they gave their approval he could amputate without delay. Her temperature after all this severe disturbance of her nervous system, generally ranged at 98½° and reached on two occasions 100°. Her pulse was habitually rapid, never falling below 108, and once reaching 136; within three weeks he had administered the same anæsthetic agent to the patient without the slightest unfavorable symptoms; he was fully satisfied that the article was pure and reliable (this was not determined by a chemist, nor is the name of the maker given, nor it is not stated that he employed the same lot as he had done before; he considers that the ether was pure because he used it out of the same can as for a patient suffering from an obscure injury of the shoulder). The method of administration was the same employed previously; a sponge placed in a cone made from a folded towel. The administration was by a resident physician who administers it in all cases in the hospital for two months, and is then replaced by another. (It is not stated how long the resident physician had used this agent). His plan he had explained to his colleagues; he states: "I introduced a steel probe through the opening to the deepest part of the abscess and made its point prominent under the skin, and with a knife rapidly cut down upon it. The incision was about two inches long. I had completed my own personal examination, and had asked my colleagues to satisfy themselves, when my attention was called by the resident giving the ether, that the movements of respiration had ceased and he could find no pulse at the wrist. Ultimately every means of resuscitation were tested, and respiration was partially restored and the pulse came up under stimulants. She was placed again upon the table and he prepared at once to amputate without the further use of ether (forgetting that she was saturated with it and in third stage). This can never be done and should not be attempted, else we lose the only chance of saving the patient, as I have before dwelt upon this subject* in both editions of my work. If the patient is exposed to cold after ether, then follow fatal symptoms, and death is a secondary effect of collapsed lungs or bronchi filled with mucus secretions. Under such circumstances the stimulants must be kept up for at least six or seven hours and the patient kept very warm, and recovery will slowly follow. If the two remaining nervous centres which supply the heart and respiration cease simultaneously, as seemed here to be the case, all motion is over and death will follow if chloroform is employed, but if ether, when it is stopped, recovery from the insensibility and prostration will invariably take place on one condition: that the body be kept warm and the system be stimulated by the rectum."

The post mortem examination was made by Dr. Walker, the pathologist of the hospital. The subject was poorly nourished; post mortem rigidity not marked. On the right arm two ecchymotic spots about the size of a silver dollar were found; another on the right side of the neck. On the right thigh, external side, was found an incision; this, when enlarged, led into a sinus and extended from the middle of the thigh as far down as the border of the condyles of the femur. The posterior surface of the femur for the space of three inches was denuded of periosteum. The walls of the sinus were covered with clotted blood. No external conditions to be noted were found. On opening the thorax, the lungs were found perfectly free from adhesions. On the lower border of both lungs were a few emphysematus blebs; the lungs were otherwise healthy. (It is not stated how this was determined, whether they pitted on pressure or were crepitant throughout. The cut surface was not described, nor was there any test by floating in water, or any notice of plugging of the vessels and if the blood was fluid. He makes no reference to the secretions in the bronchi or trachea. The pericardial cavity contained about three drachms of serum. The muscular substance and valves of the heart were appar-

*The advantages and Accidents of Artificial Anæsthesia: first ed. p. 20, second ed. p. 47.

ently normal. The spleen was slightly enlarged and thickened. The kidneys were very anæmic (no microscopic examination of them was made). There was no structural change to be found in them. The bladder was normal. The uterus showed a slight granular condition about the external os. The liver was of a brownish yellow color (no microscopic examination of a section). There was a considerable quantity of fat found in the acini. The small intestines were normal except for the anemia. In the large intestines great congestion was found extending from the sigmoid flexure to the ilocœcal valve. The mucous membrane was of a bright red color and the vessels largely dilated. No ulceration was found any where. The stomach was normal except marked aneurism. The brain was normal."

All the evidence in this post mortem and the previous history goes to show that this woman was in a pathological condition which demanded extreme caution and prudence in the employment of any anæsthetic. She had been exhausted by prolonged discharge of pus, etc., and profuse hemorrhage, and was in a state of chloro-anæmia, in which the nervous system becomes very susceptible to impressions on nervous centres, death being very apt to follow.

It is a well known fact that ether was charged with deaths in which, upon careful examination by the Boston Committee, it had no part (see abstract of report of this Committee at p. 31 of our work). And Dr. R. can only hunt up from a French work three deaths from 1847 to 1859, without even the briefest history given.

In the "*Trait D'Anesthesia Chirurgicale*," by Dr. I. B. Rottenstein, a dentist of Paris, published in 1880, he gives the relative mortality from chloroform and other anæsthetics as follows, from Prof. Andrews (Chicago *Medical Examiner*): of 200.893 cases, Sulphuric ether 1 in 23,204 administrations; Bi-chloride of methylene, 1 in 75,000; Nitrous oxide, 1 in 75,000; mixture of chloroform and ether, 1 in 5,588; chloroform, 1 in 2,733; (this table is not absolutely correct, still I give it for what it is worth.) Dr. Rottenstein reduces (p. 386) the 13 cases collected by Kappeler of Berlin, an ardent advocate of chloroform, to 4 well authenticated accidents from the effects of the *ether alone*. Compression of the carotid in an apoplectic patient aged eighty-four, death occuring two or three hours after administration, with lung complications present, mixture of amyl and ether; 9 cases in which the patients were asphyxiated with blood and vomited matters, and two cases of death from surgical shock complete the 13 cases of Kappeler. Taking Dr. Andrews' statistics and Dr. Richardson of London, we obtain up to 1872, a mortality of 4 in 92.815; or 1 in 23.204; add my 5 cases to those collected by Dr. C. Dawson, of Leeds, as having occurred from 1873 to 1878, making 18 cases in six years; then add 3 more from 1878 to 1880, with the almost universal use of this anæsthetic, and see how small the mortality has been compared with that of chloroform, which up to 1879, was 370, as in our table of deaths, and we added that there has been not less than 20 deaths since, making in all 390.

In a recent letter from Dr. I. W. Crenshaw, of Cadiz, Kentucky, he states, "The case to which you refer in your article, as having occurred in Ballard Co., Ky., should have been Burton, Marshall Co., Ky.;" and he further states, "Three cases of death from chloroform occured within a radius of fifty miles, and within a short time of each other," showing how many cases of death occur from this most powerful anæsthetic agent which never were reported.

### Mixed Anæsthetics.

When ether is mixed with chloroform (and alcohol in certain definite proportions only, see our work) it so modifies its character that the mortality is reduced, showing that the two articles are entirely dissimilar in their nature, chemical composition and effects. Yet we have proven by a series of careful experiments, the unequal rate of evaporation of the various liquids in the composition of these mixtures. If these are given by a careless person and on a towel, no matter how much starched, towards the end of the operation the patient will inhale nothing but pure chloroform.

Our reviewer says that "the danger is not near so great if given from a sponge; a squeeze now and then and a fresh addition of liquid obviates the difficulty." We doubt this. We have reported four deaths and Dr. Kappeler two, and the same authority states that he was obliged to resort to measures of resuscitation three times in two hundred administrations of these mixtures.

(To be Continued.)

